# Mating-regulated atrial proteases control reinsemination rates in *Anopheles gambiae* females

**DOI:** 10.1038/s41598-020-78967-y

**Published:** 2020-12-15

**Authors:** Priscila Bascuñán, Paolo Gabrieli, Enzo Mameli, Flaminia Catteruccia

**Affiliations:** 1 Department of Immunology and Infectious Diseases, Harvard T. H. Chan School of Public Health, Boston, MA USA; 2 Dipartimento di Medicina Sperimentale, Università degli studi di Perugia, Perugia, Italy; 3 Present Address: Centers for Disease Control and Prevention, Entomology Branch, Atlanta, GA USA; 4 Present Address: Dipartimento di Bioscienze, Università degli studi di Milano, Milan, Italy; 5 Present Address: Department of Genetics, Harvard Medical School, Boston, MA USA

**Keywords:** Proteases, Model invertebrates, Gene expression analysis, Insect hormones

## Abstract

*Anopheles gambiae* mosquitoes are the most important vectors of human malaria. The reproductive success of these mosquitoes relies on a single copulation event after which the majority of females become permanently refractory to further mating. This refractory behavior is at least partially mediated by the male-synthetized steroid hormone 20-hydroxyecdysone (20E), which is packaged together with other seminal secretions into a gelatinous mating plug and transferred to the female atrium during mating. In this study, we show that two 20E-regulated chymotrypsin-like serine proteases specifically expressed in the reproductive tract of *An. gambiae* females play an important role in modulating the female susceptibility to mating. Silencing these proteases by RNA interference impairs correct plug processing and slows down the release of the steroid hormone 20E from the mating plug. In turn, depleting one of these proteases, the Mating Regulated Atrial Protease 1 (MatRAP1), reduces female refractoriness to further copulation, so that a significant proportion of females mate again. Microscopy analysis reveals that MatRAP1 is localized on a previously undetected peritrophic matrix-like structure surrounding the mating plug. These data provide novel insight into the molecular mechanisms shaping the post-mating biology of these important malaria vectors.

## Introduction

The majority of *Anopheles gambiae* females mate only a single time in their lives^1^. Monandry in this important African malaria vector is maintained permanently for the female’s lifespan and is at least partially triggered by the sexual transfer of the male-synthetized steroid hormone 20-hydroxyecdysone (20E)^2^. During mating, this ecdysteroid, synthesized in the male accessory glands (MAGs)^3^, becomes packaged together with other seminal secretions into a gelatinous mating plug which is deposited into the female atrium^4^. Mating plug transfer in *An. gambiae* is essential for sperm storage in the female spermatheca^5^, and 20E released from the plug regulates a number of post-mating processes that are key to the reproductive biology of these mosquitoes. Besides inducing refractoriness to further mating that underlies monandry^2^, sexual transfer of this steroid hormone triggers an increase in egg development after blood feeding^4^, the oviposition of developed eggs^2^, and ensures sperm viability in the female sperm storage organ^6^.

The ability of males to form a mating plug and to synthesize 20E in their MAGs are both derived traits that have evolved in some branches of the anopheline lineage; while *Anopheles* species from Africa and Southeast Asia, belonging to the subgenus *Cellia*, coagulate their seminal secretions to form a 20E-containing gelatinous structure, other anophelines such as the Central American *Anopheles albimanus* do not^7,8^. Consequently, the key role of male 20E in regulating female’s physiology and behavior after mating appears uniquely confined to some *Anopheles* species^7^. Indeed, in the fruit fly *Drosophila melanogaster*, similar female post-mating responses are instead regulated by small accessory gland peptides (Acps) produced by the MAGs and transferred to the female during copulation^9^. Specifically, the sex peptide (SP) is a major elicitor of short- and long-term oviposition and mating refractoriness behaviors in this species^10,11^. Once transferred to the female, SP activates a specific G protein-coupled receptor (GPCR), the Sex Peptide Receptor (SPR), in neurons innervating the ovaries and uterus^12,13^, inducing profound changes in female physiology and behavior^14^. The hormone-like peptide Acp26Aa—known as ovulin—and the pheromone DUP99B also contribute to the short-term increase in ovulation and egg laying after mating^15–18^. Some of these proteins, such as ovulin, must undergo sequential proteolytic cleavage by seminal serine proteases before eliciting the post-mating response in the female^19,20^.

In *An. gambiae*, the 20E-regulated cascades mediating female post-mating responses are still largely unknown. Upon sexual transfer, the mating plug is digested in the female atrium in the space of 24–36 hours^5,21,22^, likely via the function of atrial proteases. In agreement with this hypothesis, some digestive proteases are abundantly expressed in the virgin atrium and become strongly and permanently downregulated after mating^2,23^, suggesting a specific function in the processing of seminal secretions that may become dispensable after the completion of copulation. Two of these atrial enzymes, the chymotrypsin-like serine proteases, AGAP005194 and AGAP005195, are also found in mating plug samples dissected from freshly mated females^5^, indicating a possible direct or indirect role in plug breakdown. Transcriptional repression of these genes appears to be controlled directly or indirectly by 20E, as both *AGAP005194* and *AGAP005195* are largely suppressed in the atria of virgin females injected with this steroid hormone^2^.

In this study, we show that the serine proteases AGAP005194 and AGAP005195 are specifically expressed in the atrium, where they play a major role in regulating the post-mating switch of *An. gambiae* females. Silencing their function by RNA interference results in misregulation of both mating plug digestion and the release of 20E from this gelatinous structure. Importantly, depletion of AGAP005194 (which we call here Mating Regulated Atrial Protease 1, MatRAP1) prior to a first mating event increases the remating rates of females exposed to a second male. Immunofluorescence analysis suggests that this protein is localized on a previously undetected matrix-like structure surrounding the mating plug, suggesting a possible role of this enzyme in the formation and/or function of the matrix. Altogether these data reveal novel molecular factors affecting the female susceptibility to further mating, expanding knowledge of a key reproductive process in these important vectors of human malaria.

## Results

### *AGAP005194* and *AGAP005195* are atrial-specific proteases downregulated by mating

**Figure 1:**
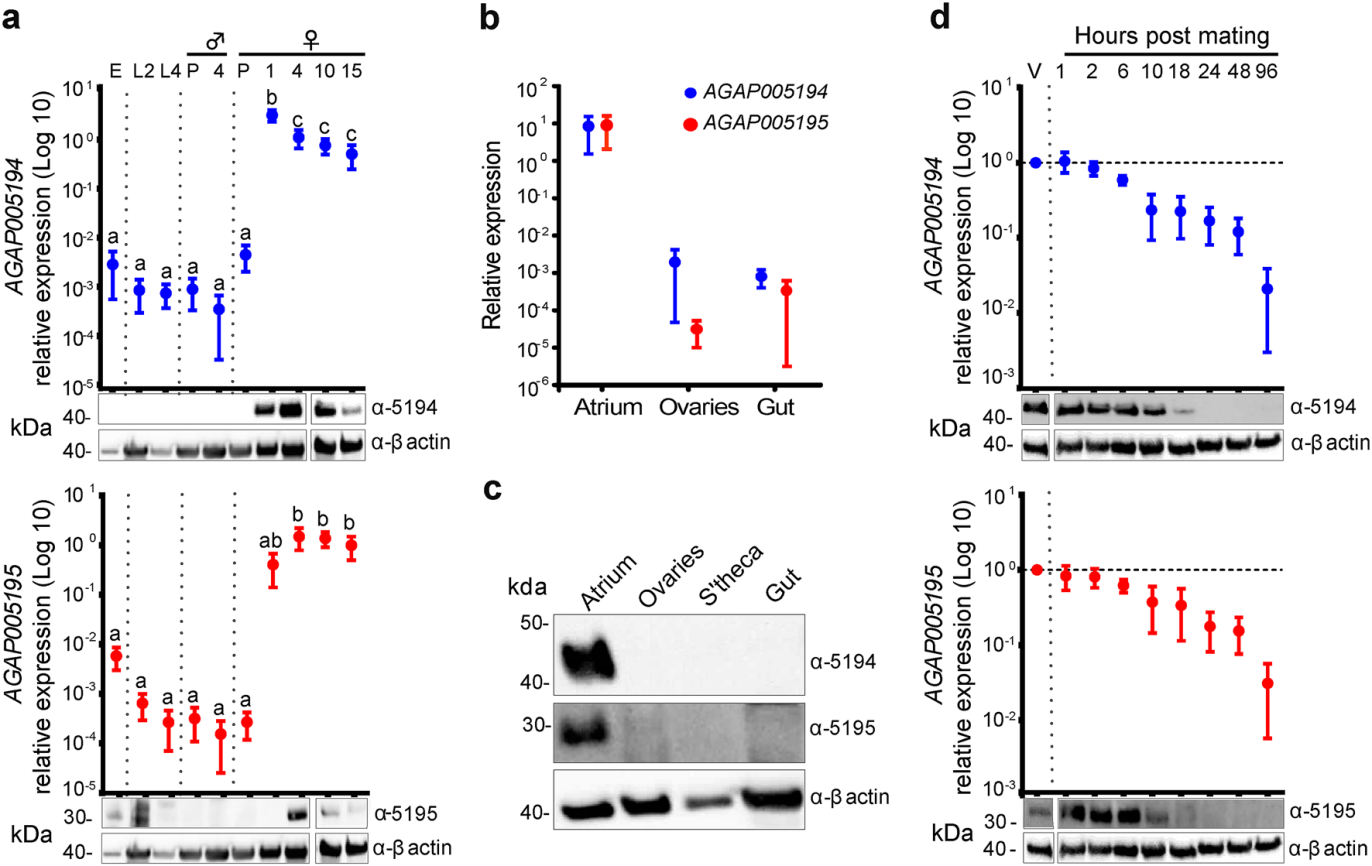
*AGAP005194* and *AGAP005195* are two atrial-specific proteases strongly downregulated by mating. (**a**) Analysis of transcript and protein levels of *AGAP005194* (upper panel) and *AGAP005195* (lower panel) in male and female mosquitoes at different time points of the life cycle: E: eggs; L2, L4: second and fourth larval instar stages; P: pupae; 4: 4 day-old adult male; 1–15: 1–15 day-old adult virgin females. Gender was not determined for embryos and larvae. (**b**) Transcript and (**c**) protein levels in different tissues (atrium, ovaries, spermatheca and gut) in 4-day-old virgin females. (**d**) Transcript and protein levels of *AGAP005194* (upper panel) and *AGAP005195* (lower panel) in females at different time points after mating. Transcript levels are relative to virgin females (V). In all panels, transcript levels were normalized against the housekeeping gene *RpL19*. Points represent the mean (± SEM) of 2–3 biological replicates each comprising 3–15 pooled samples per time point. Means with the same letter are not statistically different (*p* > 0.05). β-actin served as a loading control for the western blots. Full-length blots are presented in Supplementary Figs. 1, 2 and 4, respectively.

As an initial step towards elucidating the functions of *AGAP005194* and *AGAP005195*, we characterized their transcriptional and translational levels throughout mosquito development. These proteases shared very similar expression profiles: they were detected at low or negligible levels in juvenile stages and adult males, while expression levels were high in virgin females (Fig. 1a and Supplementary Fig. S1) (one-way ANOVA, 5194: F_(9,28)_ = 23.49, *p* < 0.0001; 5195: F_(9,28)_ = 11.8, *p* < 0.0001, Tukey test, detailed results in Supplementary Table S1), and specifically in the atrium (Fig. 1b,c and Supplementary Fig. S2). Both proteases were glycosylated—a feature critical for protein folding, secretion, stability, and, possibly, substrate recognition^24^—explaining their higher apparent molecular weight than expected (29.85 kDa for AGAP005194 and 27.5 kDa for AGAP005195) (Supplementary Fig. S3).

Transcript and protein levels steady declined after mating (Spearman's rank-order correlation, *5194*: r_s_ (27) = − 0.79, *p* < 0.0001; *5195*: r_s_ (27) = − 0.75, *p* < 0.0001), and by 24 h post mating (hpm) both proteins were below detection level, demonstrating complete suppression of expression (Fig. 1d and Supplementary Fig. S4).

### The atrial proteases regulate mating plug digestion

**Figure 2:**
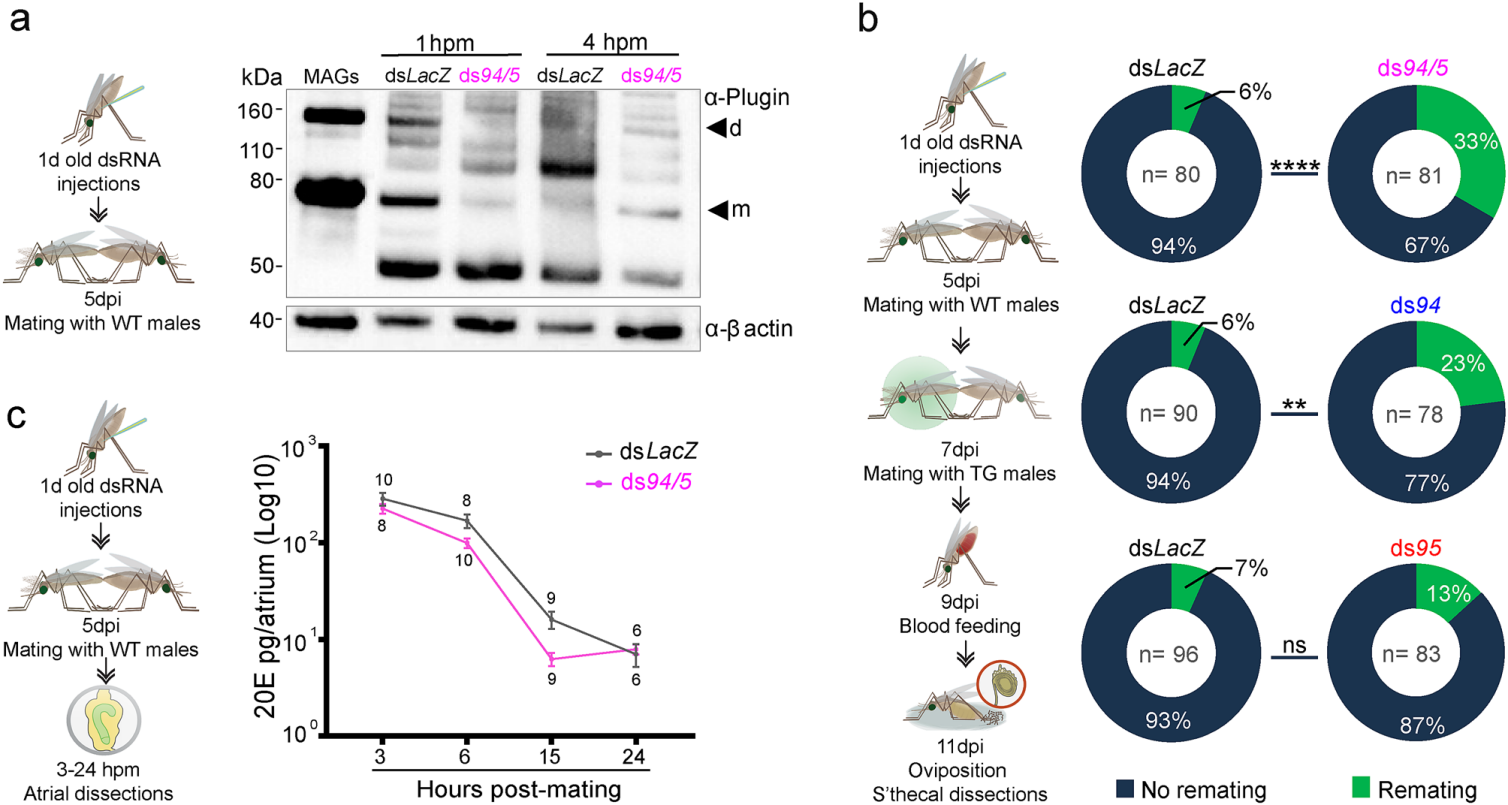
Atrial proteases affect the timely digestion of the mating plug and female refractoriness to further mating. (**a**) Processing of the abundant mating plug protein Plugin in the female atrium is accelerated over the first hours after copulation in proteases-depleted females (ds*94/5*) compared to a control group (ds*LacZ*). Male accessory gland (MAGs) extracts were used as controls for unprocessed Plugin. Arrowheads indicate the Plugin monomer (m, 80 kD) and dimer (d, 160 kD). Full-length blots are presented in Supplementary Fig. 5. (**b**) Remating rates are significantly increased in *ds94*/*5*-injected females after exposure to a second male compared to females from the control group (ds*LacZ*) (top pie charts). This effect appears to be primarily driven by the depletion of *AGAP005194* (middle pie charts) rather than *AGAP005195* (bottom pie charts). (**c**) Time-course analysis of the levels of sexually transferred steroid hormone 20-hydroxyecdysone (20E) in *ds94*/*5*-injected females reveals a faster decrease in 20E levels in the atrium than the control group (ds*LacZ*) at earlier time points after mating. Each point represents the mean (± SEM) levels of 20E in pools of three atria collected from 2 to 3 biological replicates. Numbers represent pools per treatment per time point. A schematic diagram of the experiments is provided in all panels. *dpi* days post-injection.

As AGAP005194 and AGAP005195 were previously identified by mass spectrometry in mating plug samples^5^, and their downregulation corresponds to the timing of plug digestion^5,21,22^, we investigated whether they are involved in mating plug processing. After co-silencing *AGAP005194* and *AGAP005195* by RNAi injections of double-stranded RNAs (ds*RNAs*) targeting these enzymes (ds*5194/5*, shortened to ds*94/5*), we performed a time course of digestion of Plugin, a highly abundant mating plug protein that is cross-linked to other seminal proteins via the enzymatic activity of the male-specific, plug-forming transglutaminase AgTG3^5^. Western blot analysis of plug samples using α-Plugin antibodies resulted in the classical multi-band pattern^5^, in which it is possible to recognize the monomeric and dimeric forms of Plugin (around 80 kDa and 160 kDa, respectively, Fig. 2a and Supplementary Fig. S5). Other bands were also observed, such as the catalytic product of Plugin (~ 50 kDa)^5^ present in both groups as early as 1hpm. Interestingly, at this same time point, the monomer and dimer bands were very faint in ds*94/5* females, and a band at around 100 KDa appeared, likely as a result of cross-linking between two proteolytic products. Similarly, the proteolytic product and the band at ~ 100 kDa was more evident in ds*LacZ* 4 hpm, while it almost disappeared in ds*94/5* by the same time point (Fig. 2a and Supplementary Fig. S5). Thus, silencing of the two female proteases resulted in misregulated processing of Plugin over the first hours after copulation compared to the control group, suggesting a critical involvement of the two proteases during plug digestion.

### AGAP005194 affects female remating rates, likely by slowing down the release of the male steroid hormone 20E

To assess whether timely and correct digestion of the mating plug affects female post-mating physiology, we measured the effects of *AGAP005194* and *AGAP005195* silencing, either individually or in combination, on a number of post-mating traits including oviposition (number of females laying their eggs), fecundity (number of eggs laid by each female), fertility (percentage of hatched eggs) and remating rates (percentage of females mating a second time). No differences in oviposition rates, fecundity, and fertility were observed when comparing protease*-*depleted females to controls after mating and blood-feeding (Supplementary Table S2, Supplementary Fig. S6). However, when we tested refractoriness to further mating, we found that protease-silenced females showed significantly higher remating rates when exposed to a second (transgenic) male (ds*LacZ* = 6.25%, ds*94/5* = 33.33%; Fisher’s exact test, ds*LacZ* vs ds*94/5 p* < 0.0001) (Fig. 2b, top pie charts), as verified by determining the number of females siring transgenic progeny from the second mating event. This effect was still detectable after only depleting *AGAP005194* (ds*LacZ* = 6.25%, ds*94* = 23.08%; Fisher’s exact test, ds*LacZ* vs ds*94 p* < 0.01) (Fig. 2b, middle pie charts). Remating rates also appeared higher than in controls after single ds*95* injections, however this difference was not statistically significant (ds*LacZ* = 6.67%, ds*95* = 13.25%; Fisher’s exact test, ds*LacZ* vs ds*5195 p* > 0.05) (Fig. 2b, bottom pie charts). Overall, these data show that the function of female proteases, in particular AGAP005194, affect remating rates.

The regulation of female refractoriness to further mating is at least partially controlled by the steroid hormone 20E that is transferred during copulation^2^. Specifically, it was previously demonstrated that when sexual transfer of 20E is impaired during the first mating event, remating rates increased to 33%, similar to the rate observed after *ds94/95* silencing. We therefore measured 20E titers in the atrium of mated females in the doubleknockdown group, as these had shown the strongest remating rates. As expected, the major source of 20E titers variation (89.10%) is time, as the hormonal levels steadily decline within the first 24 hpm (two-way ANOVA, F_(3,58)_ = 218.7, *p* < 0.0001). The type of treatment (ds*LacZ* or ds*94/5*) is also a source of variation (F_(1,58)_ = 86.75, *p* = 0.0118, explaining 0.92% of the total variation). Moreover, these two factors are not independent, as their interaction term is also statistically significant (F_(3,58)_ = 3.55, *p* = 0.0197), explaining 1.45% of the variation and indicating that the treatment influences 20E levels differently over time. Overall, the graph shows a more pronounced decrease of 20E levels in the atrium of ds*94/5* females after mating compared to ds*LacZ* females (Fig. 2c). While 20E levels in ds*LacZ* females are not significantly different at 3 and 6 hpm (Sidak's multiple comparisons test, *p* = 0.149), they are so in ds*94/5* females at the same time points (*p* = 0.0039), suggesting that 20E levels start declining at a faster rate in protease-silenced females early after mating. In contrast, at later time points (15 and 24 hpm) 20E levels are decreased in ds*Lacz* (*p* = 0.0124) but not in ds*94/5* females (*p* = 0.8216). This supports the idea of an earlier decline of 20E levels in the atrium of ds*94/5* females after mating. All together, these results suggest that when the proteolytic activity of AGAP005194 is impaired, mating does not induce full female refractoriness to further copulation, possibly due to faster release of male 20E from the mating plug.

### The atrial protease AGAP005194 associates with a peritrophic matrix surrounding the mating plug

**Figure 3:**
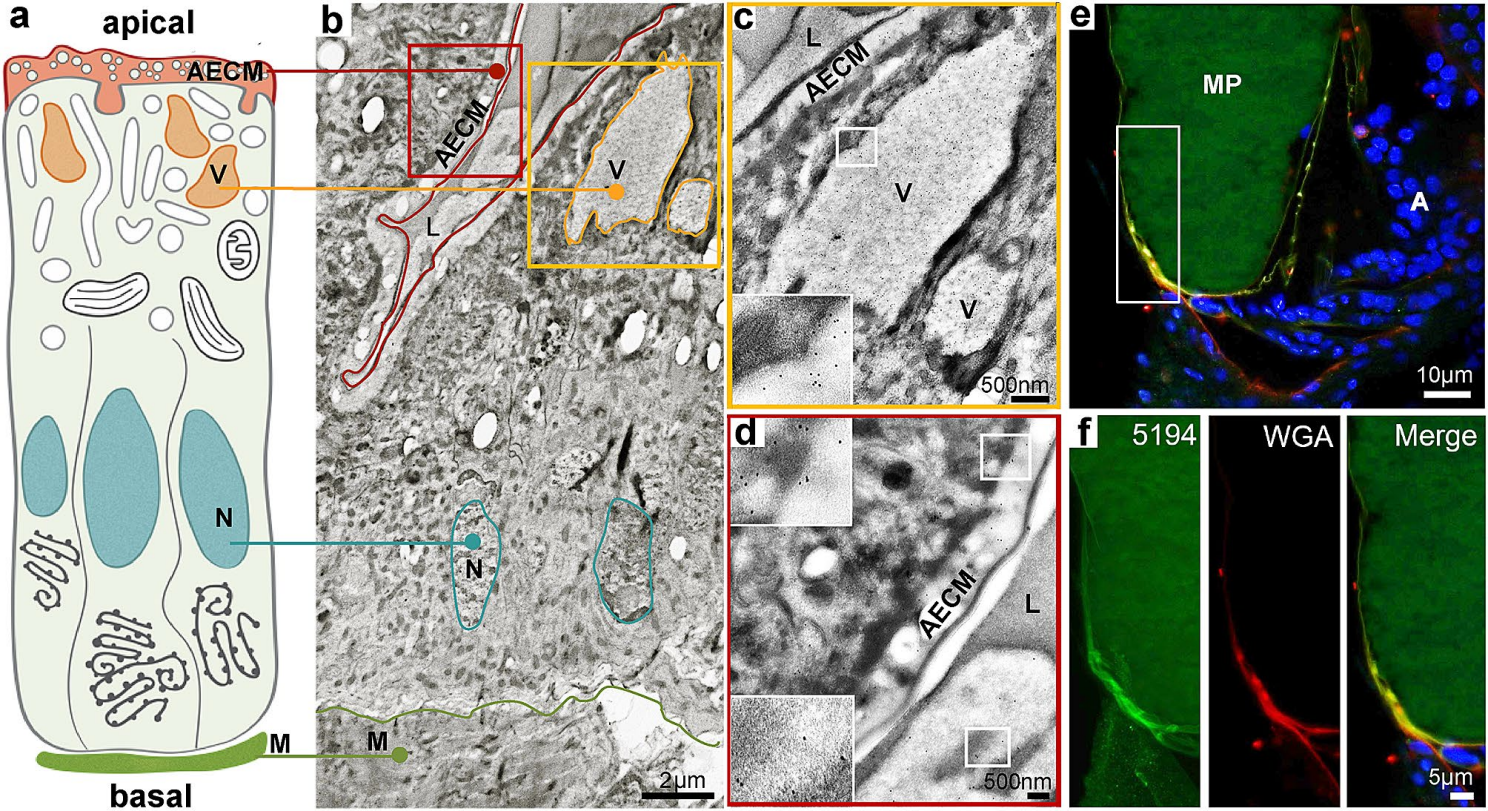
AGAP005194 is localized on a matrix-like structure surrounding the mating plug. (**a**) Graphical representation of the highly polarized atrial cells of virgin females (adapted from Rogers et al., 2008). (**b**) Immunogold Electron Microscopy of virgin atria using an AGAP005194-specific antibody. Gold particles (black dots) mainly localize in storage vesicles (V), apical extracellular matrix (AECM) and atrial lumen (L). *N* Nucleus, *M* muscle. (**c**,**d**) Higher magnification of the insets shown in (**b**). While the majority of the signal is detected in atrial cell vesicles (**c**), some signal is occasionally detected in the atrial lumen (**d**), possibly due to some secretion occurring in virgins or to mechanical disruption of atrial cells during dissection. (**e**) Immunofluorescence assay of a female atrium 3 hpm reveals that AGAP005194 (bright green) colocalizes with a thin non-nucleated (DNA, blue/DAPI) sac-like polysaccharidic structure around the plug which is also stained with wheat germ agglutinin (WGA, red). Colocalization of AGAP005194 with the chitin-binding protein WGA is notable in the membrane around the plug. *A* atrium, *MP* mating plug. (**f**) Higher magnification of the inset shown in (**e**). A faint green is visible due to the autofluorescence nature of the mating plug.

Given its prominent role in mediating refractoriness to further mating, we decided to further characterize AGAP005194 by determining its localization in virgin and mated females using AGAP005194-specific antibodies. Using immunogold electron microscopy in virgin females, we detected AGAP005194 within the apical extracellular matrix (AECM) of the highly polarized epithelium of virgin atrial cells, as well as in large vesicles localized in the apical region of the cells, which occasionally appeared to be releasing their content into the atrial lumen (Fig. 3a–d).

To determine protease localization after mating, we performed an immunofluorescence assay (IFA) shortly after copulation and detected AGAP005194 on a thin, non-nucleated envelope surrounding the plug (Fig. 3e,f), similar to the peritrophic matrix that forms around the food bolus. We also exposed these mating plugs to wheat germ agglutinin (WGA), which recognizes *N*-acetyl-glucosamine polymers present in chitin that constitutes part of peritrophic membranes. WGA was detected on the same matrix around the plug, and partially co-localized with AGAP005194 (Fig. 3e,f). No signal was instead detected in atrial cells or on the plug itself (Fig. 3e,f). These results suggest that AGAP005194 is secreted into the atrial lumen upon mating and interacts with a peritrophic matrix-like structure that surrounds the mating plug.

## Discussion

Serine proteases are a conserved group of proteins that play a role in numerous essential physiological functions including reproduction^25,26^. In *Drosophila*, sequential proteolytic cleavage of male-transferred proteins by seminal serine proteases is needed to activate the female post-mating response^19,20^. For instance, the male trypsin-like serine protease ‘seminase’ (CG10586) is required for normal processing of both the pro-hormone ovulin and the sperm-storage protein Acp36DE in the female reproductive tract, demonstrating the occurrence of specific proteolytic events involving both male and female components^19,20^. Our study identifies a proteolytic enzyme that plays an important role in the post-mating physiology of *An. gambiae* females. We show that AGAP005194 (and possibly, but to a minor extent, AGAP005195) ensures the correct processing of seminal secretions and affects the release of the sexually transferred steroid hormone 20E, with important implications for the female receptivity to further mating. The atrial specificity of AGAP005194 and AGAP005195, combined with their strong and permanent downregulation after mating, is compatible with their role in mating plug processing, which is accomplished in the female atrium within a similar timeframe. Notably, these atrial proteases are also significantly downregulated by mating in females collected from natural mating swarms in Burkina Faso, West Africa^27^, indicating that their function in post-mating responses is conserved in field *Anopheles* populations. For these reasons, we propose to name these proteases Mating-Regulated Atrial Protease (MatRAP) 1 (AGAP005194) and MatRAP2 (AGAP005195). These findings reinforce the hypothesis that virgin females are poised to receive and process vast amounts of male seminal secretions after mating^23^, an event that occurs once in their lifetime^1^.

The increase in remating events observed in ds*5194* females may be due to impaired function of the male hormone 20E, whose titers in the female atrium showed a faster decline in protease-silenced females compared to controls. Our hypothesis is that females depleted in this protease have a reduced response to male 20E signals, and possibly to other seminal secretions transferred in the mating plug. Combined with the observed misregulated digestion of the major mating plug protein Plugin, our results suggest that plug processing must be finely tuned to accomplish the timely release of 20E and trigger downstream signaling cascades associated with female mating refractoriness. Conversely, we observed no effects of protease silencing on oviposition, another post-mating response that is regulated by sexually transferred 20E^2^. While the reasons behind this lack of effect are unclear, these findings suggest that downstream signaling cascades regulating egg laying may differ from those that trigger mating refractoriness. More specifically, unlike refractoriness, oviposition and other post-mating traits may not rely on the pace at which the mating plug is digested and 20E is released. Interestingly, a recent study has shown that 20E-induced oviposition occurs via processes regulated by the stress- and immune-responsive c-Jun N-terminal kinase (JNK)^28^. In future studies, it will be important to ascertain whether JNK is involved in switching off the female mating receptivity as well and shed light on how the regulation of these two post-mating behaviors differs.

**Figure 4:**
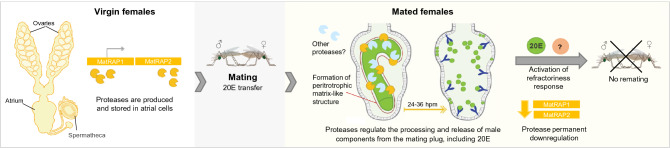
Schematic diagram of the function of atrial proteases in plug digestion and mating refractoriness. Females serine proteases MatRAP1 and 2 are produced and stored in the atrial cells of virgin females. During mating, the male ecdysteroid hormone 20E becomes packaged together with other seminal secretions into a gelatinous mating plug which is transferred into the female atrium and processed within 24–36 h postmating (hpm). MatRAP1 regulates the processing and release of these male components, and is potentially involved in the formation of a peritrophic matrix-like structure that surrounds the mating plug and may protect it from other female (or male) proteases. The correct processing of the mating plug, and the timely release of 20E trigger the refractoriness of females to further mating. Male transferred 20E also permanently downregulates the expression of MatRap1 and 2 after mating.

How does MatRAP1 affect mating plug digestion? We show that while in virgin females this protease is stored in large vesicles in the apical region of the atrial cells, shortly after mating it is localized on a previously undescribed matrix-like structure that surrounds the mating plug. This matrix may be polymerized in response to the distention of the epithelium caused by the delivery of the mating plug, as observed in the gut peritrophic matrix, which is secreted in response to physical distension of the gut after a blood meal^29,30^. This observation is compatible with the known localization of proteases of the gut of hematophagous insects, which are found on the peritrophic matrix that surrounds the blood bolus after each blood meal^31–33^. In *An*. *gambiae,* a proteomic analysis of the midgut peritrophic matrix identified abundant levels of serine proteases, in particular, trypsins and chymotrypsins, and suggested that these proteases are transiently linked with peritrophins via N- or O-linked glycans as they transit through the matrix into the midgut lumen to exert their proteolytic functions^32^. It is therefore possible that the formation of this matrix in the *An. gambiae* atrium allows the accumulation of MatRAP1 and other serine proteases that play a role during the process of plug digestion. Alternatively, and perhaps more plausibly, it is also possible that, similar to serine proteases associated with the gut peritrophic membrane, this enzyme may play a role in the formation and structural integrity of the matrix^32,34^. Matrix assembly could occur, for instance, via the activation of chitin synthesis^34^, or by the partial fragmentation of high molecular weight multi-chitin binding domain proteins like peritrophins^35^. In turn, matrix formation may protect the plug from other female (or male) proteases that would otherwise indiscriminately digest it (for a diagram of this hypothesis, see Fig. 4). This hypothesis is in agreement with the observation that while MatRAP1 was found on the matrix, it could not be detected on the mating plug itself (Fig. 3e,f). Thus, when this atrial protease is silenced, this may lead to misregulated plug digestion, resulting in a slower release of 20E and female failure to fully respond to the mating signals associated with refractoriness.

Interestingly, multiple studies in *Drosophila* have linked female post-mating biology to the evolution of post-mating, prezygotic reproductive barriers^36,37^ Moreover, mating-induced transcriptional changes are postulated to play a role in shaping the evolutionary arms race between the sexes^38–41^. *MatRAP1* and *MatRAP2* are paralogous genes clustered on chromosome arm 2L in the *An. gambiae* genome, and likely represent the product of an ancient gene duplication event pre-dating the formation of the *An. gambiae* complex^42,43^. Both proteases have been shown to be evolving under long-term and episodic positive selection, with multiple sites under selective pressure positioned near the catalytic triad and in the specificity pocket that potentially cause differential substrate binding^42,43^. Evolution of these enzymes may have therefore differentially affected the digestion of seminal fluid among anopheline species, a process associated with fertility and, in turn, speciation in the fruit fly^36,37,44,45^.

## Methods

### Mosquito rearing

Experiments were carried out in a laboratory strain (G3) of *An. gambiae* mosquitoes reared under 28 °C, 80% relative humidity, 12 h/12 h Light/Darkness photoperiod.

### dsRNA synthesis and injections

A 561 bp long region of AGAP005194 and a 232 bp fragment of AGAP005195 were amplified from cDNA of virgin females using the following primers: Fwd 5′-TCCGACAGTCAAGGAAGGTC-3′ and Rev 5′-AGTGTAGCATTTTCCGGCAC-3′ and Fwd 5′-GTTTGTGGCGTCTCTACCCT-3′ and Rev 5′-GCTGCAGTCAATATCCAACG-3′, respectively. Fragments were cloned into the pLL10 vector containing two T7 sites and ds*RNA* was synthesized with the MegaScript T7 RNA polymerase system (Life technologies). Sequences were analyzed to avoid off-target effects using the E-RNAi webserver (e-rnai.org). Females were separated from males as pupae to ensure their virgin status as adults. A single injection with 69 nl of ds*RNA* (8 μg/μl) or a co-injection with the double volume of 1:1 ratio of the two ds*RNA*s was performed into the hemolymph of 1-day-old virgin females. Injections were done into the thorax using an Injector Nanoject II (Drummond, Broomall, USA). Gene knockdown levels were analyzed by qRT-PCR in 15 and 12 independent biological replicates (single and double-silenced females, respectively) using the last segments of 5 females 5 days after ds*RNA* injections (Supplementary Fig. S7).

### Quantitative reverse transcriptase PCR (qRT-PCR)

Total RNA from eggs, larvae (instars 2 and 4), male and female pupae, and last segments of 3 days old male and 1, 4, 10 and 15 days old female adults was extracted with TRI Reagent (Invitrogen) in pools of 5–15 samples per time point and 3 independent biological replicates to check the expression of the proteases transcript during the life cycle. Total RNA from 3–5 pooled tissues (atria, ovaries, and midguts) from 2–3 independent biological replicates was extracted to analyze the proteases expression in different female tissues. Total RNA was also extracted from the last segment of 5 adult females before or after mating to analyze proteases expression in 3 independent biological replicates, as well as from the last segment of 5 virgin females 5 days after ds*RNA* injections to measure silencing efficiency. cDNA synthesis of 1 μg of RNA was obtained using M-MLV enzyme and random hexamers primers (Invitrogen). Expression was quantified using Fast SYBR-green (Applied biosystems) as described previously^23^, using the following primers: *AGAP005194* (Fwd 5′-GCATGTATGGGAGATTCTGGTG-3′, Rev 5′-GTGTACTGCTTTGAATATACCGACC-3′), *AGAP005195 (*Fwd 5′-CGCATCGATCGTGCTATAGC-3′, Rev 5′-AAGTAGTCCAACATCGTCACGAAA-3′). Primers were designed in an exon-exon junction to avoid gDNA amplification. The ribosomal gene *RpL*19 (*AGAP004422*) was used as a reference to normalize the expression levels of the genes of interest, using previously described primers and protocol^23^.

### Antibodies

Affinity-purified polyclonal antibodies were developed by a commercial supplier (GenScript Corp., Piscataway, NJ). To generate anti-AGAP005194 (shorten to anti-5194) antibody, the peptide epitope GTSPAKLQTINAPS (encompassing aminoacids 175–188 in the 272 aa long protein) was used for the immunization of mice, while anti-AGAP005195 (shorten to anti-5195) was raised in rabbits against the epitope GYISKDNKTTKITQ (position 150–163 in the 250 aa long protein). Anti-Plugin^5^ antibody was also raised in rabbits against the epitope NEHRDPQNHQLPSSC (position 268–282 in the 557aa long protein). For the immunoblotting assays a 1:1000 dilution of the primary antibodies: anti-5194, anti-5195, anti-Plugin and anti-β-actin (Sigma-Aldrich antibody produced in mouse) was used and HRP-conjugated secondary antibodies (Santa Cruz Biotechnologies) were used at a 1:10,000 dilution. For the Immunofluorescent assay, primary antibodies were diluted to a final concentration of 1.5 μg/ml, and secondary antibodies (Alexa Fluor Dyes) were used at a 1:1000 dilution. The antibodies utilized in the Immunogold Electron Microscopy were used at the following concentrations: anti-5194 was diluted 1:100, and Protein-A antibody was used at a 1:50 dilution.

### Immunoblotting

Protein extracts from eggs, larvae (2nd and 4th instar), male and female pupae and last segments of virgin females (5–15 pooled samples per point) were homogenized with a micropestle in 20 μl of PBS and a Protease inhibitor cocktail (Roche) to determine the proteases’ presence in different developmental stages. Protein extracts of atria, ovaries, spermathecae and midguts (3–10 pooled tissues each) were also collected to assess the proteases expression in different female tissues. To determine mating plug digestion patterns in silenced females and the corresponding control group, the last segment of 5 mated females (which contains the female atrium as well as the mating plug) was dissected. Plug processing impairment was determined by analyzing the digestion patterns of Plugin, the main protein component of the mating plug. MAGs extracts (n = 5) contained EDTA (250 mM) and dGTP (0.3 mM) to inhibit transglutaminase activity. A sample buffer (Invitrogen) and a reducing agent (DTT) were added to the extracts, which were heated at 70 °C for 10 min and loaded into a precast NuPAGE gel (Invitrogen) for electrophoresis under reducing conditions according to the manufacturer’s instructions. Proteins were transferred to a PVDF membrane using the XCell II Blot module (Invitrogen). Once transferred, blots were immunostained as in standard protocols^28^ with the following primary antibodies: anti-5194 and anti-5195, anti-Plugin and anti-β-actin. Bands were detected by ECL western blotting detection reagents (GE healthcare) or by Li-cor Odyssey imaging system. Reprobing with additional primary antibodies was performed after incubating membranes in stripping solution (10 mM Tris/HCl pH 6.8, 100 mM DTT, SDS 2%) at 50 °C for 30 min or using a Li-cor Stripping buffer (Li-cor Biosciences). Blots were replicated in a minimum of three biological replicates.

### Immunofluorescence assay

Reproductive tracts of 3–4-day old females were dissected 3 h post mating (hpm) in PBS solution and fixed in PBS with 4% paraformaldehyde, washed in PBS and treated with 3% hydrogen peroxide solution (in PBS) to quench autofluorescence. Samples were washed in PBS and permeabilized for 1 h in PBS with 0.2% Triton X-100, then blocked and permeabilized over night at 4 °C in PBS with 1% BSA and 0.1% Triton X-100. Samples were incubated in 1.5 μg/ml mouse anti-5194 antibody diluted in blocking buffer, washed and then stained with anti-mouse Alexa Fluor 488 (Invitrogen). Samples were then incubated with 5 μg/ml of wheat germ agglutinin (WGA)-Alexa Fluor 555 (Invitrogen) and in 1 μg/ml of DAPI (4,6-diamidino-phenylindole, Sigma-Aldrich). All staining steps were followed by washes in PBS with 0.1% Triton X-100. Tissues were then mounted in Vectashield medium (Vector Laboratories, Inc.) and visualized using a Zeiss Axio Observer inverted fluorescent microscope with ApoTome 2. Post-imaging processing was performed with Fiji software.

### Immunogold electron microscopy

The last three segments of the abdomens of 3 virgin females were chemically fixed in fresh 4% paraformaldehyde in 0.1 M Sodium Phosphate buffer pH 7.4 for 2–3 h at ~ 22 °C. After washing with PBS, tissues were infiltrated with 2.3 M sucrose in PBS containing 0.2 M glycine for 15 min. Tissues were frozen in liquid nitrogen and sectioned (80–100 nm) at − 120 °C. After screening toluidine blue-stained sections, several ultrathin sections per sample were transferred to copper grids, and then to formvar-carbon coated copper grids. Grids were floated on 1% BSA for 10 min to block for unspecific labeling and then incubate in 5 µl of anti-AGAP005194 for 30 min at RT. The grids were washed on PBS for 15 min and then transferred to 5 µl of Protein-A gold (diluted in 1% BSA) for 20 min. Protein-A was coupled to 10 nm diameter colloidal gold particles (Aurion). Samples were then washed in PBS for 15 min and finally in double-distilled water. Contrasting of the labeled grids was carried out on ice in 0.3% uranyl acetate in 2% methylcellulose for 10 min. Grids were handled with metal loops and the excess of liquid was removed by streaking on a filter paper (Whatman #1), leaving a thin coat of methylcellulose. Observations were performed in a JEOL 1200EX Trans or a TecnaiG^2^ Spirit BioTWIN mission electron microscope and images were recorded with an AMT 2 k CCD camera.

### Reproductive assays

Five days post RNAi injection (dpi) virgin females were placed with males and mating couples were individually collected in copula using established methods^23^. Mated females were transferred into a cage and blood-fed 1 day after mating. Two days after blood feeding, when eggs were developed, females were individually transferred to oviposition cups with water and filter paper. Three nights later, the filter paper was removed, and the total number of female laying eggs (i.e., oviposition rates), as well as the number of eggs laid per female (i.e., fecundity) and the ratio of eggs hatching into larvae (i.e., fertility) was recorded. For the remating assay, females were injected with dsRNA and mated as described above, and two days after mating, they were introduced for 2 nights into a cage with males of a transgenic strain carrying viable green fluorescent sperm^46^ (to distinguish the progeny derived from this second mating event). Females were then removed from the cage, blood-fed and transferred to individual oviposition cups to allow for oviposition. Fluorescence in the progeny was detected in larvae using a Nikon fluorescence stereomicroscope. The spermathecae were dissected and individually preserved at − 80 °C in 23 μl grinding buffer (10 mM Tris–HCl pH 8.2, 1 mM EDTA, 25 mM NaCl) until analysis. The gDNA extraction included a process of freeze–thaw (− 80 °C, + 40 °C) three times and 20 min of sonication or until each spermatheca was visibly open. Proteinase K was then added to each sample to a final concentration of 200 μg/ml, and samples were incubated at 37 °C for 1 h and 95 °C for 5 min to inactivate the Proteinase K. The gDNA was used for qPCR analyses using Y-specific primers (Fwd: 5′-GGATCTGGCCAAGAGGAGTA-3′ and Rev: 5′-CCCAACCAAGGTACTCTAACG-3′) to detect the presence of sperm and DSX-specific primers (Fwd: 5′-ATGGTGCGCTCCTCCAAGAACG-3′ and Rev: 5′-ACCTTCAGCTTCACGGTGTTGTGG-3′) to detect the presence of fluorescent sperm, thus, a second mating event.

### Measurement of ecdysteroid levels

Pools of three atria of either ds*LacZ* or ds*5194/5*-injected females were analyzed at different time points: 3 h, 6 h, 15 h and 24 h after mating. Samples were stored in 50 μl of methanol at − 80 °C until analysis. Tissues were homogenized and loaded into a 96-well plate pre-coated with an anti-rabbit IgG antibody for quantification of ecdysteroid levels via an Enzyme Immunoassay (EIA, Cayman Chemical), following the protocol previously described^3^. At least 6 pools of 3 atria from 2–3 biological replicates were included per time point per treatment.

### Statistical analyses

All statistical analyses were conducted using GraphPad Prism 8 (GraphPad Software Inc, La Jolla, CA USA). A one-way ANOVA, followed by a Tukey test, was performed to analyze the proteases transcript levels at the different mosquito developmental stages. To compare the transcriptional expression levels of females after mating, values were Log 2-transformed and then analyzed using a Spearman's rank-order correlation with Bonferroni correction. All transcriptional values were first normalized against the *RpL19* gene mRNA levels. Non-parametric Mann–Whitney tests were performed to compare oviposition rates, fecundity and fertility between the control and the experimental group. Differences in the number of females that failed to become refractory to a second mating were analyzed by using a Fisher’s exact test to compare single and double injections with their respective control groups. To compare ecdysteroid titers present in the atrium of mated females injected with either ds*LacZ* or ds*5194/5*, data were Log10-transformed, and a two-way ANOVA, followed by a Sidak's multiple comparisons test were performed to analyze the differences at specific time points.

## Supplementary Information

Supplementary Information.
