# Supplementary material for: Mating-regulated atrial proteases control reinsemination rates in Anopheles gambiae females
Source: Sci Rep. 2020 Dec 15;10:21974. doi: 10.1038/s41598-020-78967-y (PMC7738481; doi:10.1038/s41598-020-78967-y)
Supplement: Supplementary file 1 — Supplementary Information. [file 41598_2020_78967_MOESM1_ESM.docx]

**SUPPLEMENTARY INFORMATION**

Title: Mating-regulated atrial proteases control reinsemination rates in *Anopheles gambiae* females

Authors: Priscila Bascuñán^1,2^,^3^, Paolo Gabrieli^1,2,4^, Enzo Mameli^1,2,5^, Flaminia Catteruccia^1,2,^*

^1^ Harvard T. H. Chan School of Public Health, Department of Immunology and Infectious Diseases, Boston, Massachusetts, USA

^2^ Università degli studi di Perugia, Dipartimento di Medicina Sperimentale, Perugia, Italy

Present addresses:

^3^ Centers for Disease Control and Prevention, Entomology Branch, Atlanta, Georgia, USA

^4^ Università degli studi di Milano, Dipartimento di Bioscienze, Milan, Italy

^5^ Harvard Medical School, Department of Genetics, Boston, Massachusetts, USA

* Corresponding author: [fcatter@hsph.harvard.edu](mailto:fcatter@hsph.harvard.edu)

**Supplementary figures**

**
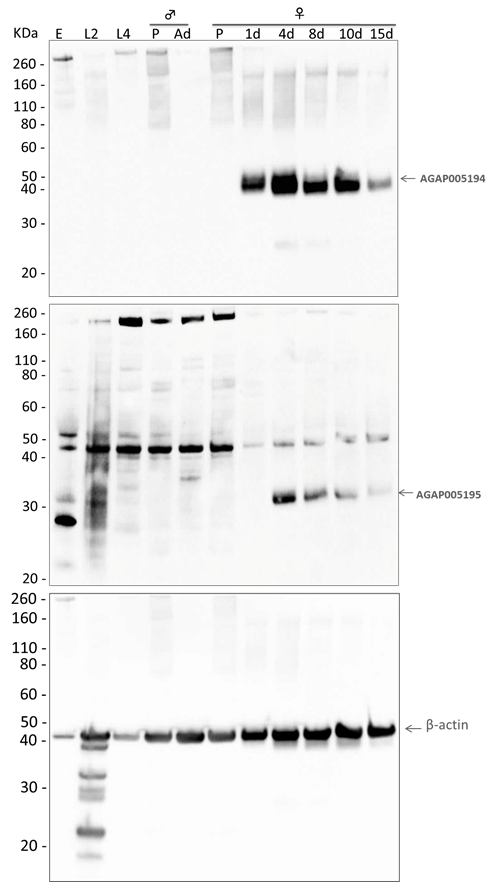
**

**Fig S1.** **Proteases expression throughout mosquito development.** Full-length western blots showing protein levels of AGAP005194 (upper panel), AGAP005195 (middle panel) and the loading control β-actin (lower panel) in male and female mosquitoes at different time points of the life cycle: E: eggs; L2, L4: second and fourth larval instar stages; P: pupae; 4: 4 day-old adult male; 1-15: 1-15 day-old adult virgin females.


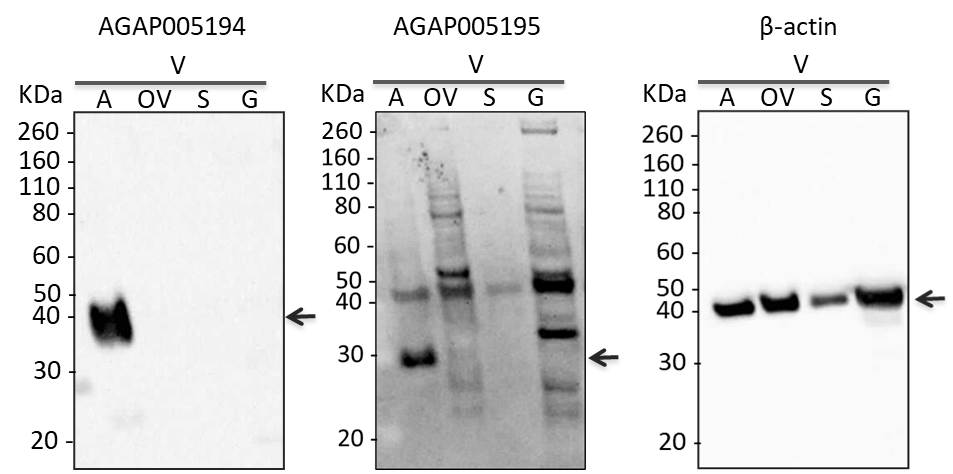


**Fig S2.** **Proteases expression in different female tissues.** Full-length western blots showing protein levels of AGAP005194 (left panel), AGAP005195 (middle panel) and the loading control β-actin (right panel) in different tissues of 4-day-old females. V: Virgin, A: atrium, Ov: ovaries, S: spermatheca and G: gut.

**
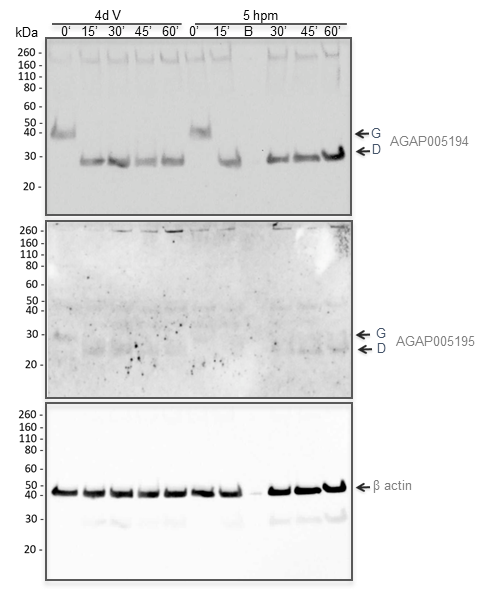
**

**Fig S3.** **Deglycosylation assay**. The presence and number of N-glycans linked to AGAP005194 and AGAP005195 were assessed by incubating atria from 4-day old virgin and mated (5 hpm) females for 15, 30, 45 and 60 min in N-glycosidase F, an enzyme that removes N-glycans, following the manufacturer’s instructions (N-glycosidase F- Deglycosylation Kit, Roche). Both proteases show a shift in molecular weight only after 15 min, indicating the presence of a single glycan chain addition. Specific anti-5194 (top panel) and anti-5195 (middle panel) polyclonal antibodies were used to detect the presence of the proteases. β-actin (lower panel) served as a loading control. G: glycosylated bands. D: deglycosylated bands. B: Blank.


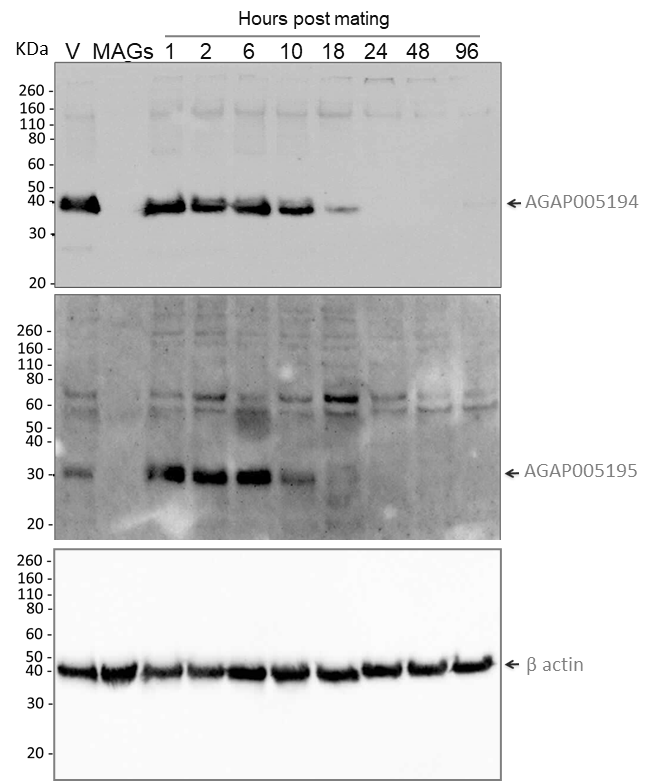


**Fig S4.** **Proteases expression after mating**. Full-length western blots showing protein levels of AGAP005194 (top panel), AGAP005195 (middle panel) and the loading control β-actin (lower panel) in females at different time points after mating (1 to 96 hpm). V: virgin females; MAGs: Male accessory glands (used as negative control).


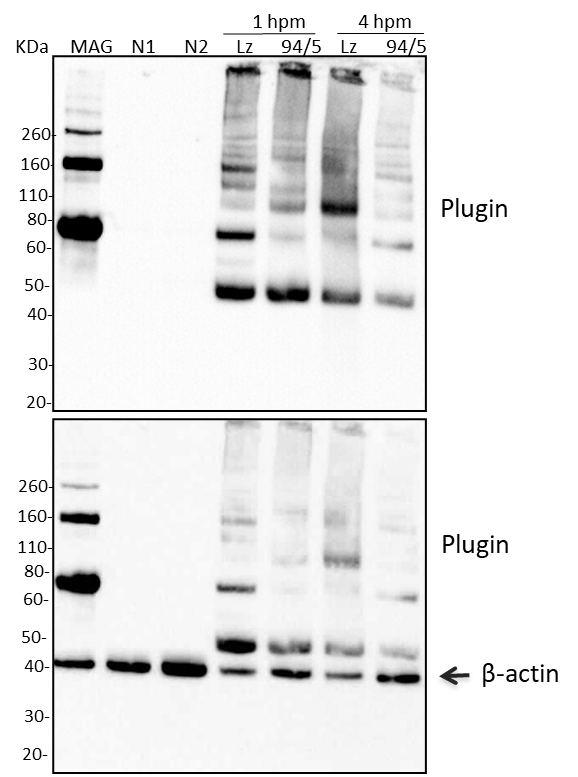


**Fig S5.** **Plugin processing in proteases-depleted females**. Full-length western blots showing protein levels of Plugin (top panel) and Plugin and the loading control β-actin (lower panel) in proteases-depleted females (ds*94/5*) compared to a control group (ds*LacZ*). Male accessory gland (MAGs) extracts were used as controls for unprocessed Plugin and virgin females were used as negative control (N1 and N2).


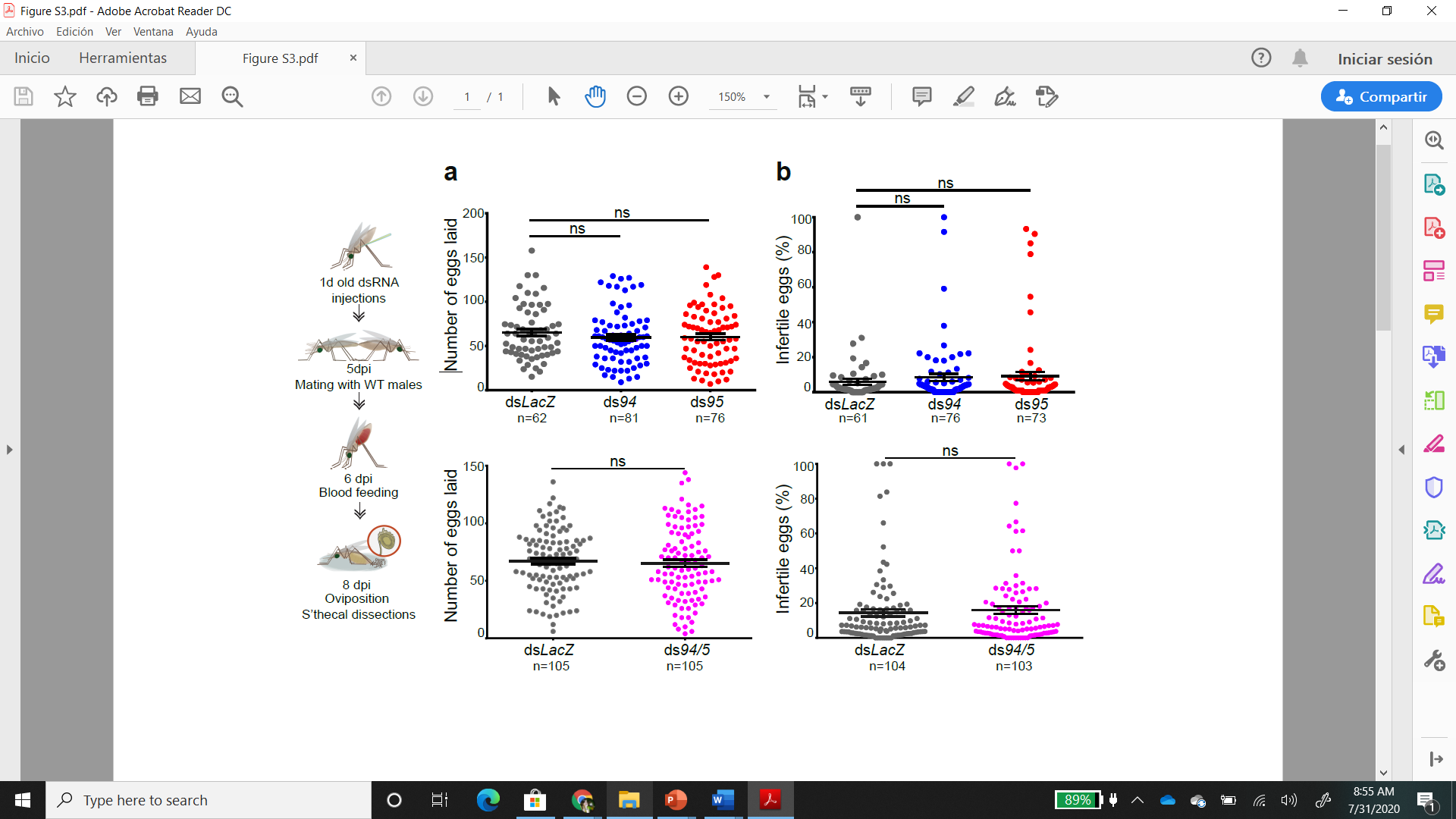


**Fig S6. Fecundity and fertility in protease-depleted females**. No differences were observed in the **a)** number of eggs laid and **b)** infertility rates in single-injected (ds*5194*, ds*5195*, top) or double-injected (ds*5194/5*, bottom) females compared to a control group (ds*LacZ*). Points represent mean (±SEM).


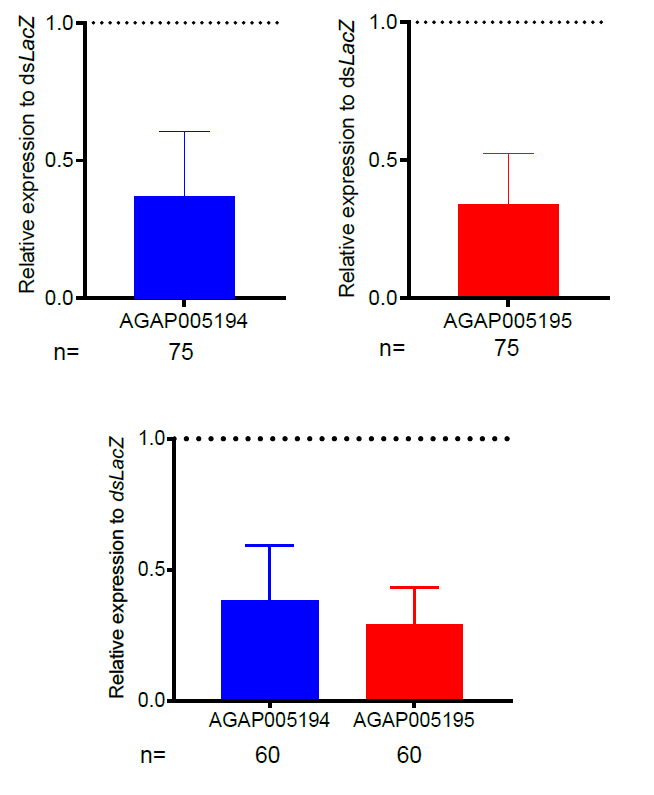


**Fig S7. Protease silencing efficiency.** *AGAP005194* and *AGAP005195* were silenced by injecting double-stranded RNAs (ds*RNAs*) targeting these enzymes either by **a)** single-injections of ds*5194* (left panel) and ds*5195* (right panel) which resulted in a reduction of 63.1 ± 23.5 % and 66.2 ± 18.7 % in the expression levels, respectively or by **b)** co-injections of ds*5194/5* that showed about 61.8% ± 20.9 % and 70.8 ± 13.8 % silencing efficiency, respectively. All transcript levels were normalized against the housekeeping gene *RpL19*. Bars show the mean ± SD of 15 and 12 biological replicates of single and double-silenced females, respectively. Each replicate represents the levels of protease transcript in the last segment of 5 virgin females 5 days after dsRNA injections.

**Supplementary tables**

**Table S1.** Post-hoc test. Tukey's multiple comparisons test of the proteases’ transcript levels at the different developmental stages. E: Eggs; L2: 2^nd^ instar larvae, L4: 4th instar larvae; P: pupae (male and female), adult mosquitoes (virgin males and females) 1, 4, 10, 15 days post-emergence.

|  | **AGAP005194** | | **AGAP005195** | | |
| --- | --- | --- | --- | --- | --- |
| **Mosquito life stage** | **Adjusted**  **P value** | **Significance** |  | **Adjusted P value** | **Significance** |
| E vs. L2 | >0.9999 | ns |  | >0.9999 | ns |
| E vs. L4 | >0.9999 | ns |  | >0.9999 | ns |
| E vs. P male | >0.9999 | ns |  | >0.9999 | ns |
| E vs. A male | >0.9999 | ns |  | >0.9999 | ns |
| E vs. P female | >0.9999 | ns |  | >0.9999 | ns |
| E vs. 1d female | <0.0001 | **** |  | 0.3618 | ns |
| E vs. 4d female | 0.0006 | *** |  | 0.0013 | ** |
| E vs. 10d female | 0.0051 | ** |  | 0.0015 | ** |
| E vs. 15d female | 0.0441 | * |  | 0.0134 | * |
| L2 vs. L4 | >0.9999 | ns |  | >0.9999 | ns |
| L2 vs. P male | >0.9999 | ns |  | >0.9999 | ns |
| L2 vs. A male | >0.9999 | ns |  | >0.9999 | ns |
| L2 vs. P female | >0.9999 | ns |  | >0.9999 | ns |
| L2 vs. 1d female | <0.0001 | **** |  | 0.2550 | ns |
| L2 vs. 4d female | 0.0005 | *** |  | 0.0008 | *** |
| L2 vs. 10d female | 0.0041 | ** |  | 0.0009 | *** |
| L2 vs. 15d female | 0.0359 | * |  | 0.0081 | ** |
| L4 vs. P male | >0.9999 | ns |  | >0.9999 | ns |
| L4 vs. A male | >0.9999 | ns |  | >0.9999 | ns |
| L4 vs. P female | >0.9999 | ns |  | >0.9999 | ns |
| L4 vs. 1d female | <0.0001 | **** |  | 0.2396 | ns |
| L4 vs. 4d female | 0.0005 | *** |  | 0.0007 | *** |
| L4 vs. 10d female | 0.0040 | ** |  | 0.0009 | *** |
| L4 vs. 15d female | 0.0356 | * |  | 0.0075 | ** |
| P male vs. A male | >0.9999 | ns |  | >0.9999 | ns |
| P male vs. P female | >0.9999 | ns |  | >0.9999 | ns |
| P male vs. 1d female | <0.0001 | **** |  | 0.2424 | ns |
| P male vs. 4d female | 0.0005 | *** |  | 0.0007 | *** |
| P male vs. 10d female | 0.0041 | ** |  | 0.0009 | *** |
| P male vs. 15d female | 0.0363 | * |  | 0.0076 | ** |
| 4d male vs. P female | >0.9999 | ns |  | >0.9999 | ns |
| 4d male vs. 1d female | <0.0001 | **** |  | 0.2333 | ns |
| 4d male vs. 4d female | 0.0004 | *** |  | 0.0007 | *** |
| 4d male vs. 10d female | 0.0036 | ** |  | 0.0008 | *** |
| 4d male vs. 15d female | 0.0319 | * |  | 0.0072 | ** |
| 4d female vs. 1d female | <0.0001 | **** |  | 0.2409 | ns |
| P female vs. 4d female | 0.0008 | *** |  | 0.0007 | *** |
| P female vs. 10d female | 0.0065 | ** |  | 0.0009 | *** |
| P female vs. 15d female | 0.0552 | ns |  | 0.0075 | ** |
| 1d female vs. 4d female | 0.0154 | * |  | 0.1976 | ns |
| 1d female vs. 10d female | 0.0019 | ** |  | 0.2254 | ns |
| 1d female vs. 15d female | 0.0002 | *** |  | 0.7603 | ns |
| 4d female vs. 10d female | 0.9912 | ns |  | >0.9999 | ns |
| 4d female vs. 15d female | 0.6222 | ns |  | 0.9826 | ns |
| 10d female vs. 15d female | 0.9880 | ns |  | 0.9896 | ns |

**Table S2. Statistical analysis on reproductive traits in single and double-injected females.** A Kruskal-Wallis test was performed to compare fecundity and fertility of single-injected females (ds*5194 and* ds*5195*) and a control group (ds*LacZ*). A Mann Whitney test was performed to compare the same traits in double-injected females (ds*94/5*) and a control group (ds*LacZ*). For oviposition rates a Chi-square test was performed for single injected-females and a Fisher's exact test was carried out for double-injected females. No statistically significant differences were observed among groups.

|  | Single injections | | | Double injections | |
| --- | --- | --- | --- | --- | --- |
| **Oviposition rates** | ds*LacZ* | ds*94* | ds*95* | ds*LacZ* | ds*94/5* |
| % | 98.39 | 93.83 | 96.05 | 80.15 | 76.64 |
| *p* value* | 0.39 | | | 0.85 | |
| **Fecundity** | ds*LacZ* | ds*94* | ds*95* | ds*LacZ* | ds*94/5* |
| Mean | 64.25 | 59.66 | 60.08 | 67.13 | 65.19 |
| Std. Error | 4.08 | 3.49 | 3.69 | 2.65 | 3.13 |
| *p* value* | 0.75 | | | 0.51 | |
| **Infertility** | ds*LacZ* | ds*5194* | ds*5195* | ds*LacZ* | ds*5194/5* |
| Mean | 5.88 | 8.44 | 9.13 | 14.35 | 15.95 |
| Std. Error | 1.76 | 2.00 | 2.44 | 2.09 | 2.13 |
| *p* value* | 0.72 | | | 0.57 | |
